# Serum Chloride and Admission Status Are Potential Prognostic Markers of High-Risk Polyps: A Prospective Characterization of Colorectal Polyps in a Tertiary Hospital in Saudi Arabia

**DOI:** 10.7759/cureus.26116

**Published:** 2022-06-20

**Authors:** Abdulrahman Algassim, Toufic Semaan, Manhal A Aldaher, Abdulsalam Alluhaydan, Ameen Jaddoh, Saeed Al-Zubide, Shakir Bakkari, Naif Benragosh, Thamer Aldarsouny, Ibrahim Alruzug

**Affiliations:** 1 Department of Gastroenterology and Hepatology, King Saud Medical City, Riyadh, SAU; 2 Department of Gastroenterology and Hepatology, Prince Sultan Military Medical City, Riyadh, SAU; 3 Department of Gastroenterology and Hepatology, King Saud Medical City, Riaydh, SAU

**Keywords:** saudi arabia, patient admission, inpatients, chloride, hypochloremia, advanced polyps, colonic polyps

## Abstract

Background: Colorectal cancer (CRC) is the most common cancer in men in Saudi Arabia. Other than age and family history, clinical and laboratory prognostic surrogates that may aid in streamlining and prioritization of screening colonoscopies are scarce. Through the examination of the local prevalence of advanced and malignant colorectal polyps, we hypothesized that the presence of certain clinical or laboratory parameters may signify an association with having high-risk polyps.

Methods: A prospective study over a period of one year starting on January 21, 2018 and involving all adult patients undergoing colonoscopy at King Saud Medical City, Riyadh. Of the total 1,104 recruited patients, 717 were included. The patients were sub-grouped based on the presence or absence of polyps. Patients with polyps were further sub-grouped into high-risk or low-risk polyps. Comparisons between groups were performed using univariate, relative risks (RRs), and multivariate analyses.

Results: Our polyp detection rate was 34.7% and our adenoma detection rate was 21.3%. The prevalence of advanced adenoma was 15.2% and the prevalence of malignant polyps was 6.7%. Several prognostic markers were associated with high-risk polyps such as advanced age (RR = 1.35, 95% confidence interval [CI]: 1.03 to 1.78), male gender (RR = 1.18, 95% CI: 1.06 to 1.31), inpatient status (RR = 1.46, 95% CI: 1.04 to 2.21), and low serum chloride (RR = 1.89, 95% CI: 1.05 to 2.37). With multivariate analysis, the hazard ratios for inpatient status and hypochloremia were 1.67 (95% CI: 1.034 to 2.612) and 1.12 (95% CI: 1.011 to 1.265), respectively.

Conclusion: We report the prevalence of malignant colorectal polyps in Saudi Arabia which was not reported before. Two unique prognostic markers for high-risk polyps were identified, namely, inpatient status and hypochloremia.

## Introduction

Colorectal cancer (CRC) is the most common cancer in men and the third most common cancer in women in Saudi Arabia [1]. The pathogenesis of CRC is largely accepted to be through the adenoma to carcinoma sequence [2]. Therefore, prevention via early detection of adenomatous polyps through colonoscopy is the standard of care [3]. The prevalence of CRC among the Saudi population has been steadily rising [1]. Consequently, optimizing local screening programs is essential. Several international guidelines have benchmarked an adenoma detection rate (ADR) of 25% in asymptomatic patients aged 50 or above [4]. New data suggest that the bar might be raised to 30% or even 35%[5]. However, in other parts of the world, the prevalence of polyps and adenoma in the same age group failed to reach these recommendations [6,7]. In Saudi Arabia, the highest reported polyp detection rate (PDR) is 24.8%[8] and the highest reported ADR is 18% [9]. Local screening practices require more data to benchmark the local ADR and PDR. Malignant polyps are defined as polyps that harbor carcinomatous cells in situ and invade the muscularis mucosa but are limited to the submucosa [10]. The prevalence of malignant polyps can reach up to 11% [11]. In Saudi Arabia, however, there are no reports on the prevalence of such polyps.

The risk of having polyps in general and advanced polyps, in particular, is linked to many factors. The best-established factors are family history of CRC, advanced age, inflammatory bowel diseases (IBD), alcohol, smoking, diet, and obesity [12,13]. All the aforementioned factors can carry a prognostic significance prior to screening. Also, the presence of these factors may hasten cancer screening in susceptible individuals. Indeed, earlier initiation of CRC screening is recommended for those with IBD or a family history of CRC and reduces cancer mortality among these groups [14,15]. For this reason, the identification and utilization of these prognostic factors will reduce CRC mortality.

Clinical and laboratory indicators for the presence of colorectal polyps are scarce. However, there are a few CRC risk calculators such as the Colorectal Cancer Risk Assessment Tool which was developed by the National Cancer Institute (NCI) [16], and QCancer prediction tools. Although the use of these prediction models has not reached its full potential, it is sometimes useful in risk estimation [17]. These calculators use parameters that are specific to the region where they were developed and cannot be used ubiquitously. For example, the NCI tool is tailored toward Whites and is not accurate for Blacks or Hispanics [16]. Therefore, risk factors that are relevant or unique to our study population must be probed.

Here, we seek to determine the prevalence, characteristics, histological features, and laboratory values for patients with polyps and advanced polyps. We aimed to build up the local data repository on the prevalence of polyps, including advanced polyps, and to aid in benchmarking local screening guidelines. Furthermore, we hypothesized that clinical parameters and laboratory values may diverge among patients with high-risk and low-risk polyps which may act as a prognostic tool. As such, we report risk factors that are associated with polyps and high-risk polyps.

## Materials and methods

Study design, inclusion, and exclusion criteria

**Figure 1:**
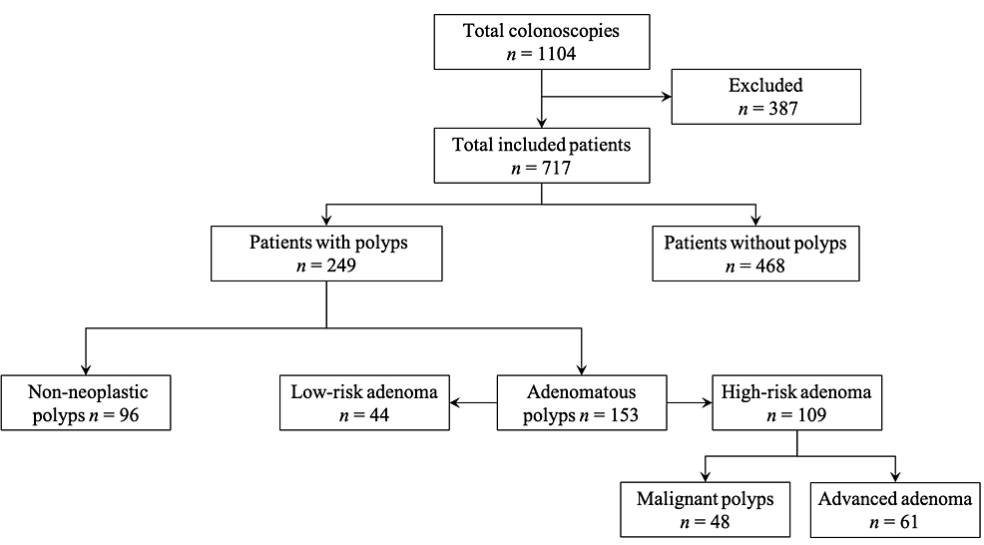
Flowchart diagram of all patients undergoing colonoscopy. Among the included 717 patients, there were 249 patients with polyps. Ninety-six patients had non-neoplastic polyps while the remaining had adenomatous polyps. Only 44 patients had low-risk adenoma and the rest had high-risk polyps. Of those, 48 had malignant polyps and 61 had other high-risk features.

The design is an observational prospective study over a period of one year starting January 21, 2018 and including all patients aged 18 and above undergoing colonoscopy in King Saud Medical City in Riyadh (Figure 1). All patients were recruited and consented to participate. The consent forms and study design were approved by the King Saud Medical City IRB (No. H1R1-10-Jan18-01). We excluded all patients with incomplete studies, which we defined as a lack of cecal intubation for any reason including poor preparation. Patients with a history of colonic polyps, malignancy, or IBD were excluded. PDR was defined as colonoscopies with polyp diagnosis and polypectomy or biopsy divided by the number of total colonoscopies. ADR was defined as colonoscopies with adenoma diagnosis divided by the number of total colonoscopies. Artificial intelligence (AI) was not used in polyps detection. Patients with polyp diagnosis alone without biopsy or histopathological reports were excluded too. We used PDR and ADR synonymously with polyp prevalence and adenoma prevalence, respectively. Patients’ demographics and laboratory data were obtained directly from the patients or the electronic health system (Medisys®, Saudi Arabia). The laboratory values investigated are hemoglobin levels, mean corpuscular volumes (MCV), mean corpuscular hemoglobin (MCH), white blood cells count, platelets count, urea, creatinine, and electrolytes. Liver function tests (LFTs) include total and direct bilirubin, alkaline phosphatase (ALP), and aminotransferases (AST and ALT). Chloride levels less than 98 mEql/L were considered hypochloremia. Admission status was either inpatient or outpatient. Patients admitted to peripheral community hospitals and referred to our center for colonoscopy were considered inpatients. Patients admitted by non-gastroenterology teams who required colonoscopy for any indication were also counted as inpatients.

Patient characterization and risk stratification

Patients were segregated into two major groups depending on the presence of polyps. Then, and by convention, we subclassified polyps into advanced, malignant, and low-risk polyps. We defined advanced polyps as having one or more of the following characteristics: any adenoma with high-grade dysplasia or villous or tubulovillous features, traditional serrated adenoma of any size, sessile serrated polyps more than 10 mm, tubular adenoma more than 10 mm, and the presence of three or more tubular adenomas [14]. Malignant polyps include adenoma with carcinoma *in situ* and invade the muscularis mucosa [10]. During analysis, both advanced adenoma and malignant colorectal polyps were clustered together as high-risk colorectal polyps. Low-risk adenomas are adenomas not having any of the aforementioned features. Non-neoplastic polyps are inflammatory and hyperplastic polyps. Low-risk adenomas and non-neoplastic polyps were clustered as low-risk polyps. Patients having more than one subclass of polyp were labeled by the polyp of the highest risk.

Statistical analysis

Statistical analysis and graphing were performed using GraphPad Prism 9.2 (San Diego, CA). Discrete variables were shown as absolute numbers or percentages and comparisons were carried out using the Chi-squared test to calculate relative risk (RR) and 95% confidence interval (95% CI). Continuous variables were expressed and graphed as mean with 95% CI. They were compared using a two-tailed student t-test when the data set was normally distributed and otherwise using the Mann-Whitney U test. Normality testing was done using D’Agostino-Pearson tests. For outlier detection, we used iterative Grubb’s and ROUT methods [18]. Regarding multivariate analysis, we applied the Cox-regression method to calculate the hazard ratio and 95 CI%. P values less than 0.05 were regarded as statistically significant and labeled as * p < 0.05, ** p < 0.01, *** p < 0.001 and **** p < 0.0001. The degree of freedom (df) for each p-value is n-2 if the t-test is the performed analysis or 1 if Chi-square is performed.

## Results

A total of 1,104 colonoscopies were performed during the study, of which 387 patients were excluded based on the aforementioned criteria. Among the 717 patients included, there were 396 males and 321 females. Seventy-nine coloscopies were performed on admitted patients and 638 procedures were performed as an outpatient (Table 1). Overall, the PDR was 34.7% (polyps detected in 249 patients) (Figure 1). The PDR in males was 39.6% (157 patients) and the PDR in females was 28.7% (92 patients). The overall ADR was 21.3% (153 patients). The ADR in males was 24.7% (98 patients) and the ADR in females was 17.1% (55 patients). Overall, 48 patients (6.7%) had malignant polyps. On the other hand, 44 patients (6.1%) had low-risk adenoma and 96 patients (13.4%) had only non-neoplastic polyps (Figure 1).

**Table 1:** General demographics of included patients. *Years

Variables		Included patients *n* = 717
Median age* (range)		49 (18-92)
Gender	Male (%)	396 (55.2%)
	Female (%)	321 (44.8%)
Admission status	Outpatients (%)	638 (89%)
	Inpatients (%)	79 (11%)

**Figure 2:**
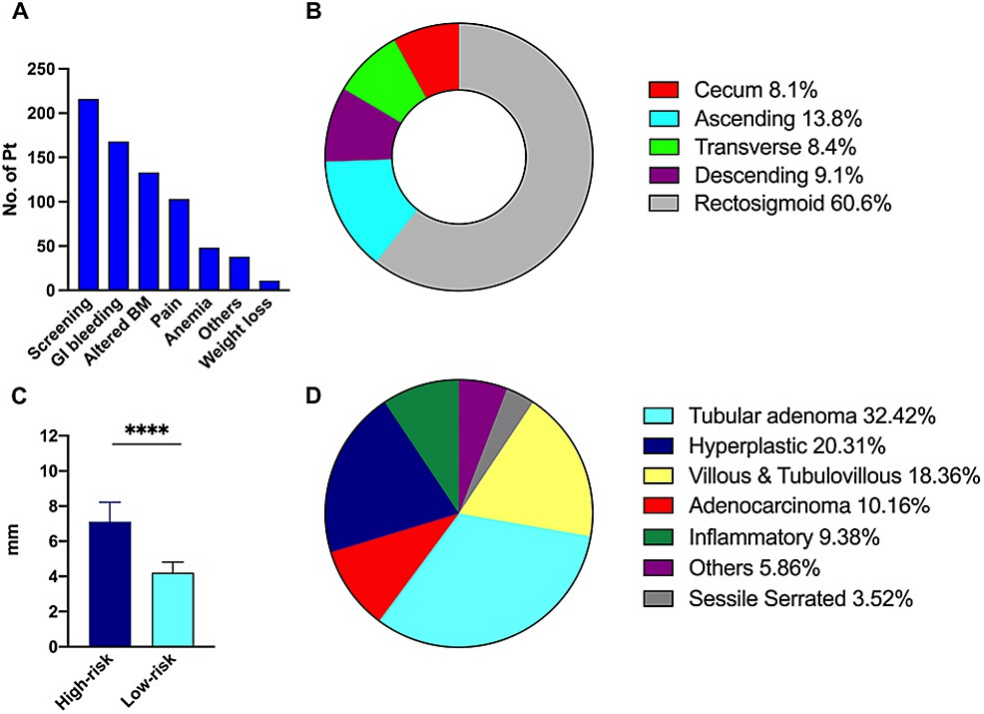
General characterization and endoscopic findings. (A) The indication of colonoscopy in descending order represented a number of patients. (B) The location of polyps detected is shown as a percentage of the total. (C) Mean diameter (millimeters) of high-risk and low-risk polyps. (D) The histological finding of the polyps removed or biopsied as a percentage of the total. ****p < 0.0001; error bars represent 95% confidence interval.

The two most common indications to perform colonoscopy were routine screening in 216 patients (30.1%) and suspected or overt GI bleeding in 168 patients (23.4%) (Figure 2A). Other causes included altered bowel motions (18.6%) and abdominal pain (14.4%) (Figure 2A). Polyps were most frequently detected in the rectosigmoid colon, followed by the ascending, descending, and transverse colons and cecum (Figure 2B). The mean polyp diameter was significantly larger in high-risk compared to low-risk polyps (7.1 vs 4.2 mm, p < 0.0001) (Figure 2C). Of the polyps biopsied, the most common histological patterns were tubular adenoma (32.4%) and hyperplastic polyps (20.3%). Villous and tubulovillous types represented 18.4%, sessile serrated adenomas represented 3.5%, and adenocarcinoma was detected in 10.2% (Figure 2D).

**Figure 3:**
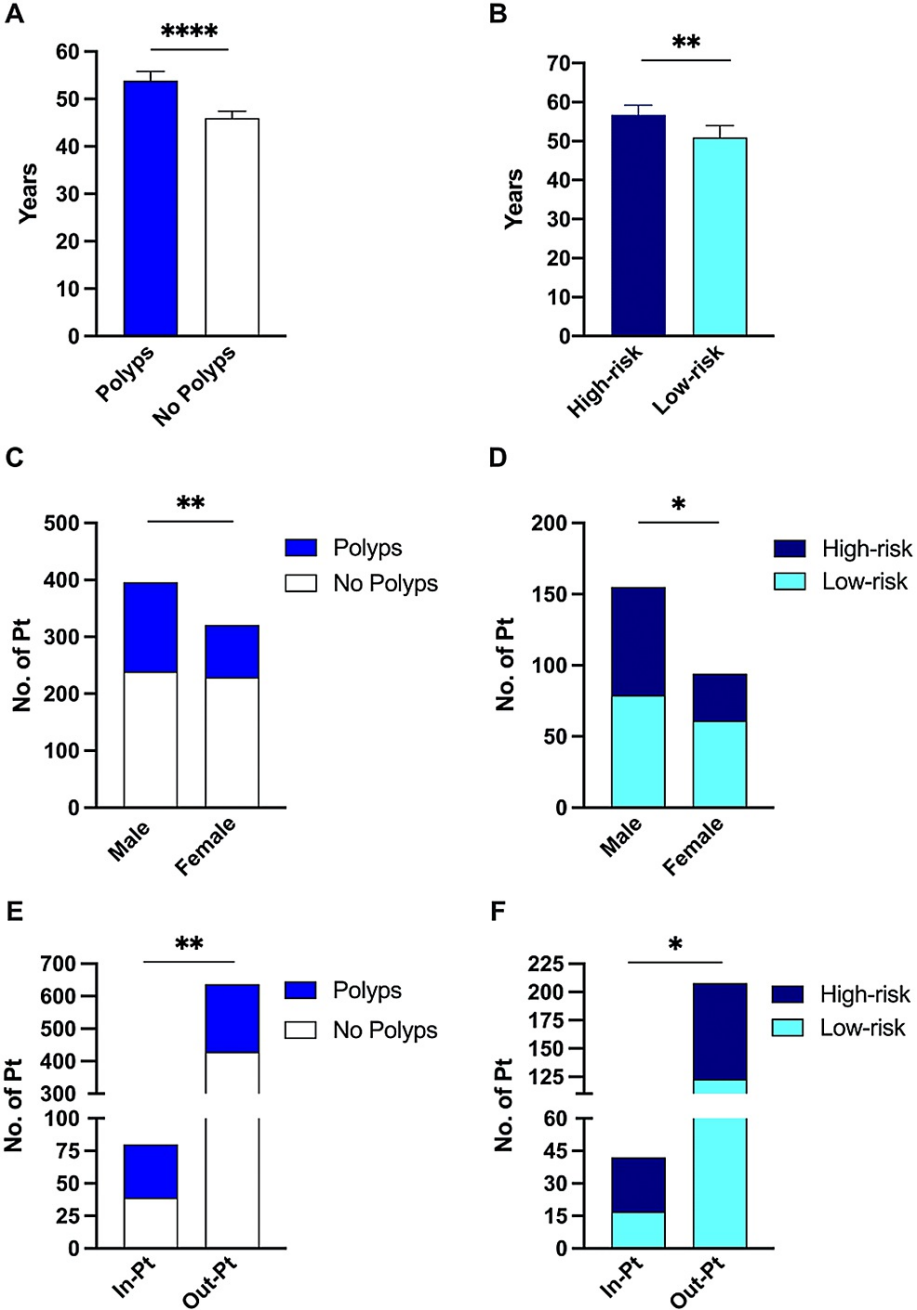
Clinical risk factors of polyps and high-risk polyps. (A) The mean age (years) of patients with and without polyps. (B) Mean age (years) of patients with high-risk and patients with low-risk polyps. (C) Stratified by gender, patients with and without polyps. (D) Stratified by gender, patients with high-risk and patients with low-risk polyps. (E) Stratified by admission status, patients with and without polyps. (F) Stratified by admission status, patients with high-risk and patients with low-risk polyps. *p < 0.05, **p < 0.01 and ****p < 0.0001; error bars represent 95% confidence interval.

The mean age of patients with polyps was significantly higher than that of patients without polyps (53.8 vs 46.0 years, p < 0.0001) (Figure 3A). Similarly, the mean age was higher in patients with high-risk compared with low-risk polyps (56.7 vs 50.9 years, p = 0.003) (Figure 3B). Stratifying the patients into four age groups, the prevalence of polyps in patients aged 60 or more was 52.1% (101 out of 194 patients). Of those, 52 patients had high-risk polyps (RR=1.35, 95% CI: 1.03 to 1.78, p = 0.036) (Table 2). Conversely, 15 patients younger than 30 years of age were found to have polyps (14.7%). High-risk polyps were found in four patients (26.7%) (RR= 0.41, 95% CI: 0.14 to 0.96, p = 0.04). In the two other age groups, namely 30-44 and 45-59, the prevalence of polyps increased with age at 25.9% and 35.6%, respectively. The same applies to the risk of having high-risk polyps, however, the RR failed to reach statistical significance (Table 2).

**Table 2:** Patients stratified by age with the prevalence of polyps and high-risk polyps in each group followed by the relative risk (RR) of having high-risk polyps. * Percentage of total cases. ^†^ Percentage of patients in the same age group.

Age group (years)	No. of Pt (%)^*^	No. of Pt with polyps (%)^†^	No. of Pt with high-risk polyps (%)	RR of high vs. low-risk polyps	95% CI	P-value
Under 30	102 (14.22%)	15 (14.7%)	4 (26.6%)	0.41	0.14 to 0.96	0.037
30-44	174 (24.26%)	45 (25.9%)	20 (44%)	0.97	0.70 to 1.44	0.899
45-59	247 (34.44%)	88 (35.6%)	33 (37.5%)	1.25	0.92 to 1.73	0.151
60 or above	194 (27.05%)	101 (52.1%)	52 (51.4%)	1.35	1.03 to 1.78	0.036

Gender was another association with colonic polyps (Figure 3C). We found that 39.65% of males had polyps compared to 28.66% of females (RR = 1.18, 95% CI: 1.06 to 1.31, p = 0.002). Furthermore, the male gender is a risk factor of high-risk as 49.03% of males with polyps had advanced features compared to 35.11% of females (Figure 3D). The male gender’s RR of having high-risk polyps was 1.273 (95% CI: 1.02 to 1.57, p = 0.03). Additionally, admitted patients undergoing colonoscopy had a higher prevalence of polyps than outpatients (50.63% and 32.76%, respectively). Inpatient status RR of having polyps was calculated at 1.38 (95% CI: 1.12 to 1.77, p = 0.001) (Figure 3E). Moreover, inpatient status was a risk factor for high-risk polyps (RR = 1.46, 95% CI: 1.04 to 2.21, p = 0.026) (Figure 3F). Using multivariate analysis and adjusting for age and gender, the hazard ratio of inpatient status for high-risk polyps was 1.67 (95% CI: 1.034 to 2.612).

**Figure 4:**
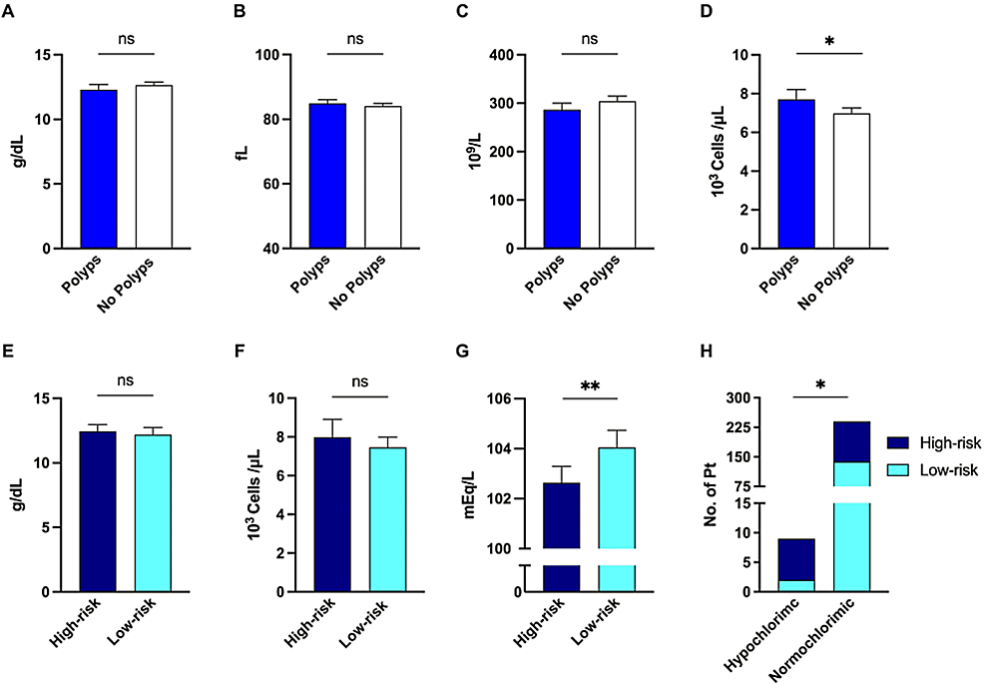
Laboratory risk factors of polyps and high-risk polyps. (A) Mean hemoglobin level in patients with and without polyps. (B) The average of mean corpuscular volume in the two groups. (C) Mean platelet count in patients with and without polyps. (D) Mean white blood cell (WBC) count in patients with and without polyps. (E) Mean hemoglobin level in patients with high-risk and patients with low-risk polyps. (F) Mean WBC count in patients with high-risk and patients with low-risk polyps. (G) Mean chloride level (mEq/L) in patients with high-risk and patients with low-risk polyps. (H) Prevalence of hypochloremia (chloride < 98 mEq/L) in patients with high-risk and patients with low-risk polyps. *p < 0.05, **p < 0.01, ns, nonsignificant; error bars represent 95% confidence interval.

Comparing patients with and without polyps using routine laboratory tests such as hemoglobin level (Figure 4A), mean corpuscular volume (Figure 4B), and platelets (Figure 4C) did not show any statistically significant difference. White blood cells (WBCs), however, were higher among patients with polyps (7.69x10^3^ cells/L vs. 6.97x10^3^ cells/L, p = 0.007) (Figure 4D). Other parameters such as urea, creatinine, LFTs, sodium, and chloride were comparable between the two groups with no significant differences (data not shown). Similarly, there was no statistically significant difference in hemoglobin level (Figure 4E) and WBC counts (Figure 4F) between patients with high-risk polyps and patients with low-risk polyps. Only one variable was statistically significant between the high and low-risk polyp groups, namely chloride levels (Figure 4G). The mean chloride level was lower in patients with high-risk polyps than in those with low-risk polyps (102.6 mEq/L vs 104.0 mEq/L, p = 0.005) (Figure 4G). Omitting outliers in both groups did not significantly change means but the difference remained statistically significant (102.6 mEq/L vs 104.2 mEq/L, p = 0.0007). Finally, the percentage of patients with hypochloremia among those with high-risk polyps was 6.42% vs 1.43% of those with low-risk polyps (RR = 1.89, 95% CI: 1.05 to 2.37, p = 0.03) (Figure 4H). With multivariate analysis and adjusting for age, gender, and admission status the hazard ratio of chloride level for high-risk polyps was 1.12 (95% CI: 1.011 to 1.265).

## Discussion

We report the highest PDR (34.7%) and ADR (21.3%) ever reported in Saudi Arabia. Despite falling short of the recommended targets of ADR, it is a step toward benchmarking local screening practices. The ADR is less than the 25% recommended target due to three main reasons. Firstly, our definition of polyp and adenoma detection excludes patients with just an endoscopic diagnosis. Even with our strict definition of detection rates, our PDR and ADR were among the highest in the region. Secondly, we included young symptomatic patients, while the value of 25% is calculated for a population aged 50 and above. The inclusion of young symptomatic patients has reduced ADR and PDR with other local investigators [8,19]. Lastly, it is reported that the prevalence of colorectal adenoma in eastern populations is less than that reported in western populations [7,20]. Compared to what has been published before, our high numbers should reflect the increasing prevalence of CRC among the Saudi population [1]. We are also the first to report the prevalence of malignant polyps in Saudi Arabia, which was 6.7% of included patients. In general, the worldwide reported prevalence of malignant polyps ranges between 0.2% and 11% [10].

The most common indication for colonoscopy in our unit was CRC screening. It is thought that GI bleeding [9,19] is the most common indication in Saudi Arabia, but this may vary from one hospital to another and between tertiary and primary centers. This paradigm shift in colonoscopy indication is encouraging and indicates the progressive efforts in Saudi screening programs and the willingness among the Saudi population [21]. Also, it may explain the progressive increase in local PDR and ADR as screening colonoscopies are becoming more common. We confirm what has been previously reported that tubular adenoma is the most common histological diagnosis of colonic polyps followed by hyperplastic polyps and tubulovillous and villous types [8]. Malignant polyps are the fourth most common histological diagnosis. Similarly, the most common location of polyps detected was in the rectosigmoid colon (60.6%). This study also emphasizes the importance of complete examinations as 8.1% of all polyps were found in the cecum. We found that high-risk polyps had a larger size compared to low-risk ones, which is consistent throughout the literature [10,11].

Age is a predictor of the presence of polyps and advanced polyps. Here, we found that young age is a protector from advanced polyps and vice versa. Half of the patients aged 60 or above were found to have polyps and half of those with polyps had high-risk ones. This confirms that age is a major risk for having polyps and advanced polyps. Likewise, the male gender is a risk factor for colonic polyps and CRC. This male preponderance is observed in both polyp prevalence in men (39.65%) vs. women (28.66%) and in the risk of having advanced polyps. This observed “gender gap” is well-documented locally [19] and worldwide [7]. Moreover, we report another predictor of having advanced polyps, namely, admission status. Admitted patients have more polyps and advanced polyps than outpatients. To the best of our knowledge, we are the first to report such an association. Inpatient status indicates that the patient is symptomatic and has an ongoing pathology related to certain morbidity. Therefore, the inpatient status is just a collective term of symptomatic co-morbidity. For that reason, adjusting for the co-morbid conditions during multivariate analysis is counterintuitive. On the same note, several groups reported that some comorbid conditions are associated with a higher prevalence of colorectal adenoma [22,23]. Nevertheless, we are the first to report the association with high-risk polyps. Some may argue that admitted patients’ higher prevalence of polyps is a result of supervised colon preparation. However, we excluded all patients with poor preparation regardless of their admission status. In any case, preparation will affect the PDR not the histological diagnosis of detected polyps. In other branches of medicine and in a similar fashion, hypercalcemia in admitted patients indicated malignancy while benign causes are expected in ambulatory patients [24]. Similarly, admission status is an indicator of poor prognosis in patients diagnosed with lung cancer, lymphoma, and uterine cervical neoplasia during their hospital stay when compared to outpatients [25-27]. These examples are abundant and widely reproduced; however, the biological mechanisms by which co-morbidities contribute to the pathogenesis of high-risk neoplasia remain to be elucidated.

Further, we searched for a dichotomy in laboratory results between patients with and without polyps. We did not find any striking difference except for the WBC count, which was higher among patients with polyps. Higher WBC counts have been associated with increased incidence and mortality in CRC [28]. We looked further for simple laboratory differences between patients with high vs. low-risk polyps. No difference was noted in the laboratory parameters between high and low-risk polyps except for chloride level. The mean serum chloride was lower in patients with high-risk polyps even after omitting outliers. Despite the stringent definition of hypochloremia, the prevalence of hypochloremia was greater in the high-risk polyp population, even if using multivariate analysis and adjusting for other variables. In spite of an extensive literature review, only one paper touched on this subject [29]. Including 5,000 CRC patients, it showed that hypochloremia is a sign of poor prognosis in terms of overall survival and disease-free survival. The authors did not show any precise mechanism of such an association; however, they claim that stress and ion channel expression in the tumor milieu might be the cause. To investigate the mechanisms is beyond the scope of our paper; however, we hypothesize that it is related to the electrolyte secretion capabilities of the adenomatous tissue. Villous adenoma-related electrolyte disturbance is well documented and it may lead to severe dehydration and acute kidney injury [30]. On the same note, the Chloride secretory capability of adenomas is proportional to its size [30]. Both large-size adenomas and villous histology are considered high-risk polyps [14]. For that reason, large villous and tubulovillous adenomas which represent a considerable part of the high-risk polyps group have chloride secretion capability which may be contributing to hypochloremia. Nonetheless, we encourage other local and international investigators to address this novel association.

## Conclusions

To the best of our knowledge, we are the first to report the prevalence of malignant polyps in Saudi Arabia. Also, we report one of the highest ADR and PDR in the region. It is very attractive to have a simple clinical or a laboratory finding that may predict the presence of polyps and carry a prognostic value. These parameters are hard to find and it is very difficult to validate. We addressed in this study two novel prognostic markers, namely, inpatient admission status and hypochloremia. Our study is limited by its relatively small sample size from a single center; thus, larger studies are needed to validate and examine the clinical relevance of our findings.
